# Research on optimization of emergency supplies customs clearance strategy from the perspective of public health security based on Chinese trade data

**DOI:** 10.3389/fpubh.2025.1520705

**Published:** 2025-01-24

**Authors:** Xia Zheng, Quan Cao, Xin Zhou

**Affiliations:** ^1^Department of Customs and Public Administration, Shanghai Customs College, Shanghai, China; ^2^Department of Science and Technology, General Administration of Customs of the People’s Republic, Beijing, China

**Keywords:** public health emergencies, emergency supplies, k-means algorithm, RFM model, customs supervision

## Abstract

**Background:**

This study is significant for improving the accuracy of Customs’ cross-border supervision of emergency supplies and ensuring the timely clearance of these essential goods.

**Methods:**

To ensure both the convenience and security of Customs oversight regarding emergency supplies, this study first systematically collects and organizes representative data on the import and export trade of these supplies. Proposed an enhanced Recency Frequency Monetary (RFM) cluster analysis model, building on the principles of k-means clustering. Subsequently, the model is employed to cluster the import and export trade data of emergency supplies. Finally, the paper offers optimization suggestions for customs clearance supervision based on the analysis results.

**Results:**

The study primarily focuses on the collection and organization of import and export trade data across six major categories of representative emergency supplies. By employing K-means clustering techniques, the research develops an improved RFM cluster analysis model, referred to as TR-TF-TV, and subsequently proposes strategies for customs supervision of emergency supplies. By integrating K-means clustering techniques, this study develops an advanced RFM cluster analysis model, referred to as TR-TF-TV. It subsequently proposes customs supervision strategies for emergency supplies. These strategies include a clustering analysis of trade data to ensure safe and efficient customs clearance, the preservation of integrity and stability within the emergency supplies supply chain, the enhancement of the national emergency management system, and the improvement of response capabilities to public health emergencies.

**Conclusion:**

This analysis of trade data concerning the import and export of emergency supplies, based on the enhanced RFM clustering model, represents an exploratory initiative in original model design. Recognizing the inherent limitations associated with the model’s index design and data sample selection, we intend to refine these elements in future research, aiming to improve and validate the model to further optimize related countermeasures.

## 1 Introduction

In recent years, the global public health landscape has been marked by the potential for outbreaks and cross-border transmission of infectious diseases. Notable examples include the Ebola virus and the Middle East Respiratory Syndrome, alongside the emergence of new infectious diseases. Concurrently, traditional infectious diseases such as yellow fever and plague have re-emerged, posing significant challenges to the healthy development of international public health initiatives. These diverse infectious disease events not only present formidable obstacles to global health efforts but also exert a profound impact on public health at national ports of entry (1). The 2020 global outbreak of COVID-19 serves as a pertinent example of how frequent and complex trade exchanges between economies can exacerbate the spread of an epidemic. As individuals and goods continued to circulate, the virus expanded in both strength and speed. This situation has acted as a real-time stress test for the emergency management systems and capabilities of various economies in their responses to public health emergencies (2). On January 31, 2020, the World Health Organization (WHO) declared the outbreak of the new coronavirus a public health emergency of international concern (PHEIC). Subsequently, on March 11, 2020, the WHO characterized the outbreak as a ‘global pandemic’ (3). As of February 2022, the outbreak resulted in over 421 million infections globally, leading to approximately 5.78 million deaths.

Article 22 of the International Health Regulations (2005) explicitly states that the Customs Service is designated as the competent authority at a country’s ports of entry. This designation is crucial for interrupting the introduction and spread of infectious diseases and for maintaining public health and safety at these points of entry. Consequently, various countries maintain national security and safeguard public health in alignment with the unique characteristics of their respective governmental regulatory functions. First, taking the United States as an example, the U.S. National Security Strategy presents a comprehensive overview of U.S. policy positions regarding national security. It emphasizes the necessity for U.S. responses to defend against weapons of mass destruction, address biological weapons and pandemics, and enhance border controls (4). The U.S. Border Protection Agency, in conjunction with the U.S. Port Health Stations, build partnerships for disease surveillance and control, aims to limit the introduction and spread of infectious diseases in the United States. In order to effectively prevent public health emergencies, China Customs has systematically built and improved the port emergency management system.

The rapid spread of the epidemic around the world has brought major challenges to the implementation of effective external epidemic prevention at ports. Taking China Customs as an example, in response to the ever-changing epidemic situation, the General Administration of Customs and its directly affiliated customs authorities have steadfastly adhered to the Party Central Committee’s overarching strategy of “preventing imports from abroad and preventing rebounds at home” as well as the general policy of “dynamic clearing” for epidemic prevention and control. They have implemented the most comprehensive, stringent, and thorough measures in history, established a robust port prevention and control system, fortified the port quarantine defense line, and created a full-chain joint prevention and control closed-loop management system at the “overseas-country-door,” thereby effectively safeguarding public health. Based on the needs of COVID-19 prevention and control, the customs inspects 100% of the health declarations of all entry and exit personnel and implements strict inspections. For confirmed cases, suspected cases, symptomatic individuals, and close contacts, prevention and control measures—such as transfer, isolation, and observation—must be implemented in accordance with the requirements of the joint prevention and control mechanism. Implement boarding quarantine and strict disinfection on all ships, flights, cars and other means of transportation coming from countries or regions with severe epidemics. Imported items, especially fresh cold chain foods and their packaging, must be sampled for COVID-19 nucleic acid sampling and source control implemented. This will inevitably lead to more customs clearance links, longer customs clearance times, increased customs clearance costs, and reduced customs clearance efficiency. During peak customs clearance periods, there may even be problems such as stranded personnel, vehicle congestion, and backlog of goods. To effectively address the challenges posed by the epidemic, it is essential to identify effective countermeasures that seamlessly integrate epidemic prevention and control with customs clearance facilitation. This approach enables us to achieve both ‘prevention’ and ‘rapid clearance,’ thereby fundamentally mitigating the epidemic’s impact on customs operations. By adopting this strategy, Customs can fully leverage its role in enhancing foreign trade, fostering scientific and cultural exchanges, and facilitating the smooth flow of domestic and international dual circulations. Furthermore, this presents an opportunity to enhance the scientific rigor, accuracy, and effectiveness of Customs’ responses to various public health emergencies, while continuously improving the port’s emergency support mechanisms and optimizing the cross-border trade business environment.

The prevention and control of epidemics at ports, where Customs safeguards national security, exhibits several distinctive characteristics. These include uncertainty regarding the approval of emergency materials, time constraints in the supply and demand of resources, a diverse array of participating stakeholders, and heightened scrutiny from international public opinion. Consequently, it is crucial to investigate methods for implementing hierarchical and classified supervision of emergency supplies. This approach aims to enhance the efficiency of customs clearance and approval processes while establishing a comprehensive, all-encompassing customs emergency management system. In this context, this study initially employs an enhanced RFM model that incorporates K-means clustering as its foundation. It systematically gathers and organizes representative import and export trade data for emergency supplies from 2019 to 2022, subsequently conducting clustering analysis on this data. The findings offer strategic recommendations for Customs to implement hierarchical and classified supervision of emergency materials, effectively balancing the two critical supervisory objectives of ensuring both ‘manageability’ and ‘expeditious’ customs clearance for these materials. This approach holds significant theoretical relevance.

## 2 Literature review

### 2.1 Emergency management system for public health emergencies

In conjunction with a summary of advanced emergency management experience, public crisis events can be categorized into several distinct types: natural disasters, accidental disasters, public health emergencies, social security emergencies, and economic crises (5). In the context of medical research, public health emergencies have historically been classified into four primary phases: the onset of symptoms, acute episodes, the continuation phase, and recovery (6). Public health emergencies can be categorized into two main areas: those related to public health and those arising from health emergencies caused by infectious diseases, as well as biological, chemical, and other harmful agents. These emergencies may be mismanaged, leading to the potential for localized transmission to escalate into national or even cross-border outbreaks (7). The 4R Emergency Management Theory emphasizes the development of an integrated management system that encompasses the entire process of crisis recognition, risk assessment, early warning, monitoring, and restoration of normalcy. This theory also highlights the importance of incorporating social pluralism in the participation of key stakeholders in governance. By embodying systematic thinking in emergency management, it offers a scientific foundation for the emergency management practices of governmental departments (8). As the name suggests, emergency management explores how to rationally prevent and effectively respond to risks (9). A comprehensive review of numerous public health emergency cases reveals opportunities to transcend conventional approaches, thereby facilitating innovation in social institutions and operational mechanisms (10).The primary objective of emergency management plans is to minimize the risks associated with emergencies and to establish preemptive response arrangements based on early warning and assessment of potential threats (11). But given the typical uncertainty of emergencies (12), to better align with the unique characteristics of the emergency management process, particularly the transboundary aspects across various dimensions of time and space, it is essential to investigate strategies for ensuring and enhancing the dynamism of the plan. This will facilitate the development of a guidance process that allows for continuous updates (13). The Government must incorporate the crisis into its daily management practices by establishing a permanent, authoritative, and well-defined coordination unit (14). In the context of institutional innovation, it is crucial to emphasize the significance of involving multiple stakeholders and fully engaging non-governmental organizations. Their active participation is essential to maximize the benefits of social impact (15). This necessitates a departure from the traditional approach of passive, singular responses by the government during emergencies. Instead, it advocates for the development of a comprehensive management system that emphasizes timely responses, unified dispatch, and decentralized coordination. This system should be grounded in a full-process emergency management model, where government departments take the initiative and actively engage citizens, enterprises, non-governmental organizations, and other social groups (16). In a normalized social environment, it is essential to move beyond a passive approach to mobilizing grassroots activities, which often relies on legislation and administrative coercion. Instead, there is a need to invigorate grassroots engagement through the transformation of sectional mobilization. This shift is crucial for achieving genuine participation from various stakeholders in mass prevention and control efforts (17). To effectively address various emergencies and enhance the efficiency of emergency management, it is essential to establish emergency management institutions that facilitate the coordination of power dynamics at both local and central levels, as well as between different local entities. This coordination should be approached from both horizontal and vertical perspectives (18).

It is essential to establish an emergency management mechanism that encompasses accurate early warning and prevention measures, swift and timely responses during incidents, and robust resilience and recovery capabilities thereafter. To begin with, it is imperative to address the current low level of informatization and to implement intelligent early warning systems driven by advanced information technology (19), building information technology architecture based on high cohesion and low coupling (20), the development of adaptable early warning and drill programs for emergencies of varying categories and intensities is essential for improving intelligent decision-making in emergency management, particularly from a top-level design perspective (21). Secondly, to tackle the fundamental issues of resource scarcity and inadequate communication among governments, which are particularly prevalent during the disposal phase of events (22), mitigating a range of secondary issues, including public opinion, which may arise from inadequate management of the situation (23), there is an urgent necessity for the primary emergency management department to develop and reinforce emergency connections with both horizontal and vertical government entities (24). To effectively address the challenges associated with emergency management, it is essential to comprehensively summarize existing issues and dynamically optimize the rectification processes. This includes enhancing the transparent supervision of emergency funds, consolidating the implementation of personnel insurance, and strengthening the intervention strategies related to psychological factors (25). Additionally, there is a need to continuously improve the legal mechanisms governing emergency recovery, which can be achieved by promoting or reshaping the safety culture education among the public (26). It is essential to clarify the law from the perspectives of right boundaries, duty definitions, and resource guarantees in order to effectively implement emergency management. This clarity helps prevent issues such as self-contradiction, procedural non-conformity, and inconsistencies in scale, which can hinder the advancement of emergency management efforts during implementation (27). These challenges are indicative of a long-term and pressing concern (28).

### 2.2 Characteristics of emergency supplies for public health emergencies

A public health emergency typically refers to a highly destructive outbreak that has the potential to cause, or may already be causing, the widespread transmission of an epidemic. This characterization is largely due to the unpredictable nature and sudden onset of such events (29). In the context of emergency management for public health crises at all stages—before, during, and after an event—the foremost priority is to secure the supply of essential materials, particularly medical emergency supplies. This is crucial to ensure the organization’s capacity to deliver timely warnings, respond effectively, manage the situation, and facilitate recovery from such incidents (30, 31).

Risk factors exhibit complex, intertwined, and superimposed characteristics in the context of current sudden events. Consequently, the characteristics of emergency supplies should be examined through various classification frameworks. These include: categorization based on different uses, which primarily encompasses management, rescue, repair, and protection; classification according to varying degrees of emergency response levels, including conventional, medium, and emergency levels; differentiation based on usage categories, which are mainly divided into general-purpose and emergency response supplies; and categorization based on the types of emergencies addressed, which includes public health events, nuclear, biological, and chemical responses, as well as food poisoning incidents (32). The emergency supplies designated for addressing public health emergencies exhibit several key characteristics: the necessity for proper storage, restrictions based on category, unpredictable demand, and the imperative of timely supply (33). Therefore, it is crucial to establish and maintain emergency material stockpiles to ensure a swift response during the pre-disposal phase following emergencies. This approach guarantees the effectiveness and comprehensive coverage of the supply of essential materials (34).

### 2.3 Customs supervision for public health emergencies

Customs administrations worldwide, as key actors positioned at borders, play a pivotal role in global supply chains and in ensuring their sustainability (35). A crucial factor in ensuring the security and stability of the supply chain for emergency supplies during public health emergencies is the efficiency and safety of the import and export customs clearance process. The WCO has issued several tools and instruments to assist the customs community in enhancing supply chain management and promoting sustainability. Additionally, it has recently published guidelines to combat the COVID-19 pandemic. In response to this, numerous customs administrations worldwide are prioritizing supply chain sustainability in their strategic frameworks. However, the COVID-19 pandemic has imposed additional burdens and pressures on both customs administrations and businesses (36). The prevention and control of public health emergencies should involve implementing appropriate measures that facilitate international trade in goods and the cross-border movement of people, while ensuring compliance with public health requirements. This approach should avoid extreme measures such as closures and restrictions (37). Consequently, enhancing the supervisory capacity of the Customs Service in managing the customs clearance of emergency supplies, as well as establishing a secure, efficient, and coordinated customs clearance system, are essential strategies (38). The rapidly increasing demand for emergency supplies has shifted to the international market, particularly highlighted by the pneumonia epidemic of 2020, which constituted a public health emergency (37). Most suppliers and producers of emergency supplies encounter risks associated with disruptions in supply chains and capital flows. Furthermore, in contrast to standard trade commodities, anti-epidemic emergency supplies exhibit unique characteristics, including specific trade orders and designated materials (38). These supplies encompass a wide variety of types and forms of storage, alongside diverse and unpredictable demand patterns, as well as uncertainty regarding demand information. Consequently, there are significant challenges related to customs clearance and supervision. As a result, the demand information for China’s anti-epidemic emergency supplies is characterized by a pronounced level of uncertainty compared to that of ordinary trade commodities (39). Additionally, it is imperative to establish an emergency supplies system that guarantees collaborative supervision throughout the supply chain of these critical resources. The core element of the supply chain for emergency supplies, which is essential for maintaining security and convenience, is ‘urgency.’ It is crucial to consider the demand points for multimodal transportation and other channels (40), while maximizing the value of supplies within the constraints of limited time. This approach ensures efficient coordination and consistent operation throughout the entire process, including production, storage, sales, transportation, acceptance, and utilization. The goal is to achieve an organic unity between the convenience and security of Customs supervision within the supply chain of emergency materials. Despite the existing laws and regulations providing a foundational basis for effective implementation, there remains significant potential for urgent improvements in China’s customs emergency management. For instance, the State Border Sanitary and Quarantine Law, enacted in 1987, primarily reflects concepts and principles derived from the International Health Regulations of 1969. Consequently, there is a notable lag in its prevention and control measures for infectious diseases when compared to the updated requirements outlined in the International Health Regulations of 2005 (38). The International Health Regulations (2005) (hereinafter referred to as ‘IHR’) should focus on port activities to improve the risk assessment of public health emergencies, response measures, and disposal methods. Despite the significant importance of these core capacities for international public health, the State Border Sanitary and Quarantine Law has undergone three substantial amendments from 2007 to the present, none of which have addressed this issue (1). In addition, government departments are explicitly mandated to develop emergency response plans for significant events within their respective fields or industry sectors, in accordance with the Measures for the Administration of National Emergency Response Plans (41). In response to the new Crown Pneumonia epidemic, during the control phase of external anti-importation and internal anti-exportation at ports, the Customs Department implemented a series of measures aimed at ensuring efficient customs clearance and risk prevention for emergency supplies. However, most of these measures were temporary, acute, and reactive in nature, leading to a policy landscape characterized by fragmentation, significant overlap, poor connectivity, and low integration. Consequently, the Customs Service needs to enhance its overall mechanism and develop long-term strategies for customs clearance and the protection of emergency supplies and other goods at ports. This improvement is essential to strengthen the systematic and proactive approach to customs clearance and the safeguarding of emergency supplies (42). The U.S. Immigration and Customs Enforcement (ICE) proposes public health risk prevention and control countermeasures in the context of the spread of the new coronavirus (COVID-19) through data processing and simulation of the model (43). The Korea Customs Service has developed a digital customs system grounded in risk management guidelines and practices. This system effectively mitigates public health risks associated with the entry and exit of goods and individuals by providing quantitative data on customs operations and risk management (44). The Jordan Customs findings revealed that big data analytics can play a vital role in overcoming supply chain disruptions and enhancing the sustainability of supply chains during the COVID-19 pandemic (45). Indian Customs has implemented Information and Communication Technology (ICT) solutions to effectively respond to emergencies, ensure uninterrupted supply of essential and emergency medical supplies during blockades, and ensure that 24/7 clearance operations are successfully maintained (46).

This comprehensive analysis of the response to public health emergencies under the customs supervision of emergency supplies, in relation to the theory of the status quo, highlights the complexity and volatility of the current international trade environment, where various risks remain unpredictable. Customs, as a crucial gateway at the national frontier, must fully implement the overarching security concept of the country, thereby assuming the vital responsibility of safeguarding the nation’s borders. This involves efficiently establishing robust defenses at ports to manage a wide range of public health emergencies while ensuring the well-being of the community as a whole. Furthermore, prioritizing the life, health, and property safety of society is essential, necessitating effective management of the clearance processes for emergency supplies at ports. Building on this foundation, the study advances existing research in two key areas: firstly, it seeks to integrate data analysis with decision-making processes, adopting customs supervision as a lens through which to examine import and export data of relevant emergency materials during public health crises. This approach aims to provide a scientific basis for innovative customs supervision strategies regarding the clearance of emergency materials. Secondly, the study innovates data analysis methodologies by proposing an enhanced RFM clustering model that incorporates k-means clustering techniques for data processing and analysis. This development not only broadens the research scope of the traditional RFM model but also offers a novel theoretical tool for advancing customs supervision strategies related to the clearance of emergency materials.

## 3 Methodology

### 3.1 Define the research object

The emergence of the COVID-19 pandemic has had profound implications for public health, resulting in widespread transmission, significant economic losses, and numerous human casualties. In response, governments at all levels worldwide have implemented stringent measures to mitigate the crisis, prompting considerable concern across various sectors of society. This study focuses on the background of the COVID-19 outbreak, which began to spread globally in 2020. In February 2020, the China-WHO Joint Expert Group on New Crown Pneumonia arrived in Hubei to conduct a comprehensive, multi-dimensional analysis of the situation. Their focus was on the effectiveness of epidemic prevention and control measures, the medical treatment provided, and the availability of necessary supplies. In the first half of 2020, during the pre-spreading phase of the COVID-19 public health emergency, the Party Central Committee and the State Council made decisions and deployments in accordance with China’s Emergency Response Law and other relevant regulations. They uniformly elevated the emergency response level for the prevention and control of the COVID-19 outbreak to a first-class provincial-level response for major public health emergencies across the nation. The review of the response period during the Hubei epidemic in China underscores the critical need to enhance the capacity of the national medical emergency material industry chain, revealing significant deficiencies in the supply of emergency medical materials. To strengthen the medical material emergency protection system, the central government has introduced a series of policy documents aimed at bolstering the capacity for public health emergency material protection. Notably, the ‘Implementation Program on Improving the Public Health Emergency Material Protection System’ is among these key initiatives. A public health emergency with the following typical characteristics: (1) The global public health crisis triggered by the outbreak of COVID-19 represents a coherent and multifaceted emergency. This crisis is characterized by the airborne transmission of the SARS-CoV-2 virus, which poses significant cross-border challenges, uncertainties, and threats to public safety. Furthermore, the various issues arising during governmental crisis management are amplified through multiple information channels, highlighting both the media’s role and the international dimensions of the outbreak. Consequently, the COVID-19 pandemic exemplifies public health emergencies of its kind and necessitates collaborative efforts from global governments and the public to achieve the common objective of epidemic prevention. (2) The supply of materials during the emergency management response to the COVID-19 pandemic involves the participation of various stakeholders, including domestic governments, non-governmental organizations, businesses, individuals, and foreign governments. Consequently, the emergency material supply chain is inherently political and diverse. Given its characteristics of timeliness and complexity, it is essential to ensure the convenience and safety of the cross-border trade supply chain for emergency supplies. This situation serves as a typical case study for research on optimizing customs clearance strategies from the perspective of customs supervision. (3) The impact of epidemic events is characterized by a prolonged cycle, extensive reach, significant demand for materials, and a diverse range of material types. The statistics on the import and export trade of emergency materials, published by the General Administration of Customs of China, serve as a comprehensive data source for cluster analysis. These statistics are notable for their variety, substantial volume, authenticity, and accessibility, aligning well with the defining features of big data.

### 3.2 Define the research methods

The establishment of an effective public security mechanism within the framework of public security governance serves as a vital instrument for the execution of the government’s administrative, economic, and social responsibilities, particularly from the viewpoint of responsible governance. This approach represents a theoretically innovative research methodology in the field of public management, enabling the formulation of scientifically informed and precise management optimization decisions through the application of clustering analysis on big data in relevant domains. This paper focuses on optimizing customs clearance strategies for emergency supplies, primarily through clustering analysis of import and export trade data related to these supplies. Before conducting clustering for various material types, it is essential to apply the RFM model to assess the value of different types of emergency supplies. This assessment highlights their significance in epidemic prevention and control, thereby safeguarding the stability of social and economic order, as well as addressing the urgency of international trade and security.

The RFM model is a widely recognized and classic quantitative analysis tool utilized to assess both the current value and potential value of an object (47). The three important measures in this model are R, F, and M. The R value reflects the level of social demand associated with the measurement object, a smaller R value indicates a greater social demand, suggesting that the object is more urgently needed by the public. The *F* value represents the social stickiness of the measurement object, a larger F value signifies higher social stickiness, implying that the object is likely to maintain continuous public demand. Finally, the M value denotes the socio-economic contribution of the measurement object, a larger M value correlates with a greater socio-economic contribution, necessitating the implementation of more personalized management strategies for this category.

This paper utilizes the RFM model to conduct a comprehensive value analysis of the data. It employs the K-means algorithm to perform clustering analysis on data points exhibiting similar or identical values, thereby enabling the formulation of precise and personalized regulatory strategies. K-means, the most widely used clustering algorithm, partitions a set of N objects into K clusters, where K is a predetermined number. This algorithm operates with linear time complexity and is characterized by its rapid convergence to a local minimum, effectively representing the average of the objects within each cluster (48). The fundamental principle of the algorithm is to minimize the overall error by representing various types of samples through the mean of k clusters, achieved through iterative processes. This principle is encapsulated in two key aspects: first, the aim is to ensure that the clusters themselves are as homogeneous as possible, while also maximizing the distinction between different clusters. Second, the algorithm operates under the criterion of minimizing the sum of squared errors, with its cost function defined in Equation 1.


(1) $$J\left(c,\mu \right)=\overset{k}{\underset{i=1}{\sum }}\|x^{\left(i\right)}-\mu_{c^{\left(i\right)}}\|{}^{2}$$

In the equation above, $\mu_{c^{\left(i\right)}}$ represents the clustering mean of the ith cluster. As the similarity among the samples within each cluster increases, the squared error between the samples and the cluster mean decreases. When the number of clusters is denoted as *k*, the optimality of each cluster can be assessed by calculating the sum of the squared errors derived from all clusters. The specific steps of the algorithm are outlined as follows (Table 1).

**Table 1:** Algorithm core iteration process.

Step1: Randomly select k cluster centroid points
Step2: Repeat the following process until the algorithm converges { for each sample i, calculate the class it should belong to: $c^{\left(i\right)}:\arg\min_{j}\|x^{\left(i\right)}-\mu_{j}\|{}^{2}$ for each class j, recalculate the centroid of the class: $\mu_{j}:\frac{\sum_{i=1}^{m}1\left\{c^{\left(i\right)}=j\right\}x^{\left(i\right)}}{\sum_{i=1}^{m}1\left\{c^{\left(i\right)}=j\right\}}$ }

### 3.3 Data acquisition and processing

#### 3.3.1 Data objects

This study takes the medical emergency supplies urgently needed during epidemic prevention and control as the main data retrieval and research objects. It can be divided into six categories, each of which corresponds to a specific type of medical supplies and the corresponding HS code as shown in Table 2. Emergency supplies are classified into distinct subcategories according to their function, type, material, and intended use, each of which corresponds to specific HS codes.

**Table 2:** Medical emergency supplies categories and the harmonization system code (HS) (49).

Epidemic prevention medical emergency supplies	Categories	Name	HS Code
Medical equipment	Ventilator	Invasive ventilator	90,192,010
		Non-invasive ventilator	90,192,020
	Oxygenator	Oxygen production capacity> = 15,000 cubic meters/h	84,196,011
		Other oxygen concentrators	84,196,019
Medical protection	Mask	Mask type 1	63,079,010
		Mask type 2	63,079,000
	Protective gowns		62,101,030
	Protective glasses		90,049,090
	Gloves	Medical Plastic Gloves	39,262,011
		Medical rubber gloves type 1	40,151,200
		Medical rubber gloves type 2	40,151,900
Medical device	Forehead thermometer		90,251,990
In vitro diagnostics	Antigen reagents		30,021,500
	Nucleic acid reagents		38,220,090
Therapeutic medicine	COVID-19 vaccine		30,022,000
Sterilization products	Disinfectant	Ordinary type	38,089,400
		Medical or laboratory	84,192,000

#### 3.3.2 Data acquisition

This study utilizes data from the Customs Statistics Online Query Module available on the official website of the General Administration of Customs of the People’s Republic of China. The search parameters were specifically defined to encompass the period from November 2019 to May 2022. The analysis focused on import and export types, categorized as per their respective classifications. Additionally, the trade mode was restricted to general trade, and the currency was specified as Renminbi. The commodity codes were aligned with the HS codes for emergency supplies as outlined in Table 2. Consequently, the retrieval process yielded import and export trade data for each category of emergency supplies.

#### 3.3.3 Data processing

To ensure data homogeneity and address issues of redundancy, defaults, and other special circumstances, the research focused on the retrieval of data obtained through preliminary cleaning and merging. This process yielded the following results: (1) the data retrieval cycle for emergency supplies import contained a total of 11,178 valid entries; (2) the data retrieval cycle for emergency supplies export contained a total of 49,000 valid entries.

### 3.4 TR-TF-TV indicator design in improved RFM model

#### 3.4.1 Indicator design

The three important index designs in the TR-TF-TV model improved based on the RFM model are:

TR: trade recency, this indicator is utilized to assess the robustness of trade memory concerning emergency supplies and the extent to which customs can effectively supervise these commodities. It will inform the regulatory strategies that customs may implement for emergency supplies, including the frequency of regulatory inspections and the intensity of efforts to stimulate trade in these critical items.

TF: trade frequency, this indicator is utilized to serves as a crucial indicator for assessing the urgency of demand for emergency supplies. It forms the foundation for classifying various types of emergency supplies and informs the structure of emergency supplies trade. This understanding is essential for guiding the allocation of regulatory resources, developing regulatory strategies, and formulating customs clearance regulatory plans.

TV: trade value, this indicator serves as a critical metric for assessing the scale of trade and the significance of emergency goods in maintaining the security and stability of cross-border trade. This evaluation informs the classification of emergency goods into two categories: those subject to key regulation, characterized by high economic impact and elevated trade risk, and those governed by regular regulation. By differentiating between these categories, customs authorities can implement tailored facilitation measures, thereby achieving the regulatory objectives of stimulating trade activity and ensuring trade security.

#### 3.4.2 Indicator concepts

TR: This metric indicates the interval between the most recent trade of emergency supplies and the prior transaction. A larger TR value signifies a longer duration without transactions for emergency supplies, which increases the likelihood of dormant trading activities and potential trade losses. This scenario suggests that certain emergency supplies, particularly those of significant importance, may benefit from the activation of trade activities through convenient regulatory measures. Conversely, a smaller TR value indicates that there are imminent cross-border trade activities involving emergency supplies.

TF: Indicates the frequency of cross-border trade in emergency supplies over a specified period. A larger Trade Frequency (TF) value indicates a higher frequency of such trade, highlighting the critical nature of these supplies. Emergency supplies are not only essential for humanitarian efforts but also represent significant material commodities that contribute to the economic dynamics of cross-border trade. As their economic contribution increases, so do the associated trade risks, necessitating enhanced investment in customs supervision resources to ensure efficient and safe clearance of these materials. Conversely, a smaller TF value suggests a lack of activity in the trade of emergency supplies, which may be influenced by competition from other trade partners. For categories of emergency supplies characterized by low TF values but high trade volumes, it is imperative for customs authorities to implement targeted trade competition strategies to enhance the international market share of these essential commodities.

TV: Indicates the trade volume of emergency supplies. It serves as a significant indicator of demand for these materials and reflects the degree of trade restrictions imposed. According to Pareto’s principle, 80% of a country’s trade income is derived from a small percentage of high-value transactions, with 20% of commodities yielding the majority of this revenue. Consequently, emergency supplies that exhibit large trade volumes are deemed critical, as they represent commodities with heightened risks. Therefore, it is essential for customs authorities to implement targeted risk prevention and control measures for these high-volume emergency supplies.

### 3.5 Cluster analysis based on improved RFM model

#### 3.5.1 Preparation

This study employs the Python programming language to conduct a category analysis of emergency supplies. Initially, data preparation is essential. The required customs trade statistics can be obtained from the official website of the General Administration of Customs, specifically by retrieving the customs statistics data associated with the HS Code for emergency supplies, as detailed in Table 2. The analysis focuses on the import and export trade data of emergency supplies from November 2019 to May 2022, examining various types of emergency supplies based on the parameters of ‘data year and month,’ ‘commodity code,’ and ‘RMB’ within the dataset. The findings from this analysis contribute to a more scientific discourse regarding customs supervision strategies for diverse categories of emergency supplies.

#### 3.5.2 Data merging and extraction

The data collected during the preparation phase are stored in various Excel tables. Consequently, it is necessary to merge the data prior to extraction. Following this, the relevant data for the analysis of emergency supplies is extracted, specifically focusing on the “data year and month,” “commodity coding,” and “RMB.” The trade imports merged data file generated during this process comprises a total of 11,178 valid entries, while the trade export merged data file contains 49,000 valid entries.

#### 3.5.3 Data exploration analysis

This step primarily focuses on identifying missing data and anomalies within the dataset related to the emergency material classification TR-TF-TV model to analyze data patterns effectively. The merged data table, as previously obtained, should be analyzed further using the Describe () function in Python. This analysis will help in assessing missing data and data anomalies within the dataset. Additionally, it will calculate various statistical metrics, including the count of non-null values, maximum and minimum values, average, unique values, median, mode, variance, and other relevant results.

## 4 Results

### 4.1 Calculate TR-TF-TV value

To calculate the TR-TF-TV value, the data needs to be further processed to remove the null values of TR and the data with TV of 0. Imports and exports are obtained in Tables 3, 4 respectively.

**Table 3:** Emergency supplies trade import TR-TF-TV data.

HS Code	TR	TF	TV
30,021,500	1,121	17,993	3.61E+10
30,022,000	42	19,072	3.75E+09
38,089,400	1,126	17,988	4.3E+09
38,220,090	1,158	17,956	1.59E+10
39,262,011	392	18,722	1.2E+08
40,151,200	20	19,094	2.28E+08
40,151,900	989	18,125	9.15E+09
62,101,030	860	18,254	2.27E+09
63,079,000	2,231	16,883	6.99E+09
63,079,010	108	18,896	42,594,097
84,192,000	446	18,668	1.02E+09
84,196,011	1	19,113	365,333
84,196,019	47	19,067	18,588,676
90,049,090	1,101	18,013	5.7E+08
90,192,010	38	19,076	1.77E+08
90,192,020	45	19,069	2.59E+08
90,251,990	1,452	17,662	3.54E+09

**Table 4:** Emergency supplies trade export TR-TF-TV data.

HS Code	TR	TF	TV
30,021,500	4,268	15,128	8.64E+10
30,022,000	1,019	18,377	1.03E+11
38,089,400	2,753	16,643	2.02E+10
38,220,090	4,093	15,303	1.99E+10
39,262,011	3,095	16,301	3.83E+10
40,151,200	469	18,927	1.77E+09
40,151,900	4,387	15,009	3.48E+10
62,101,030	4,835	14,561	8.56E+10
63,079,000	6,028	13,368	4.38E+11
63,079,010	825	18,571	1.16E+10
84,192,000	3,694	15,702	2.2E+09
84,196,011	301	19,095	2.78E+08
84,196,019	2,473	16,923	5E+09
90,049,090	5,363	14,033	1.83E+10
90,192,010	283	19,113	1.69E+08
90,192,020	396	19,000	7.44E+08
90,251,990	4,708	14,688	1.74E+10

### 4.2 Data conversion

The primary objective of data conversion is to transform data into a suitable format that fulfills the requirements of data analysis and data mining algorithms. Consequently, the trade import TR-TF-TV data and the trade export TR-TF-TV data, obtained in the preceding steps, must undergo separate standardization processes. The standardized values for both the trade import and export TR-TF-TV data are presented in Table 5.

**Table 5:** Emergency supplies import and export trade TR-TF-TV data standardization processing results.

Trade Type	TR	TF	TV
Import	−0.701348	0.705615	3.427055
	0.954357	−0.936910	−0.134368
	−0.709021	0.713226	−0.073490
	−0.758124	0.761939	1.201961
	0.417289	−0.404117	−0.533264
	0.988116	−0.970400	−0.521375
	−0.498797	0.504676	0.459859
	−0.300849	0.308303	−0.296701
	−2.404623	2.395330	0.222700
	0.684289	−0.836440	−0.541819
	0.334427	−0.321914	−0.434232
	1.017271	−0.999323	−0.546465
	0.946685	−0.929299	−0.544460
	−0.670669	0.675170	−0.483830
	0.960492	−0.942999	−0.527004
	0.949754	−0.932343	−0.517977
	−1.209262	1.209485	−0.156589
Export	−0.699355	0.699355	0.328418
	0.939764	−0.939764	0.486959
	0.064962	−0.064962	−0.303138
	−0.611068	0.611068	−0.306397
	−0.107577	0.107577	−0.130319
	1.217239	−1.217239	−0.479376
	−0.759391	0.759391	−0.164439
	−0.985406	0.985406	0.320989
	−1.587274	1.587274	3.684752
	1.037637	−1.037637	−0.385306
	−0.409772	0.409772	−0.475213
	1.301995	−1.301995	−0.493589
	0.206222	−0.206222	−0.448562
	−1.251782	1.251782	−0.321131
	1.311076	−1.311076	−0.494627
	1.254067	−1.254067	−0.489143
	−0.921335	0.921335	−0.329877

### 4.3 Clustering of emergency supplies

**Figure 1:**
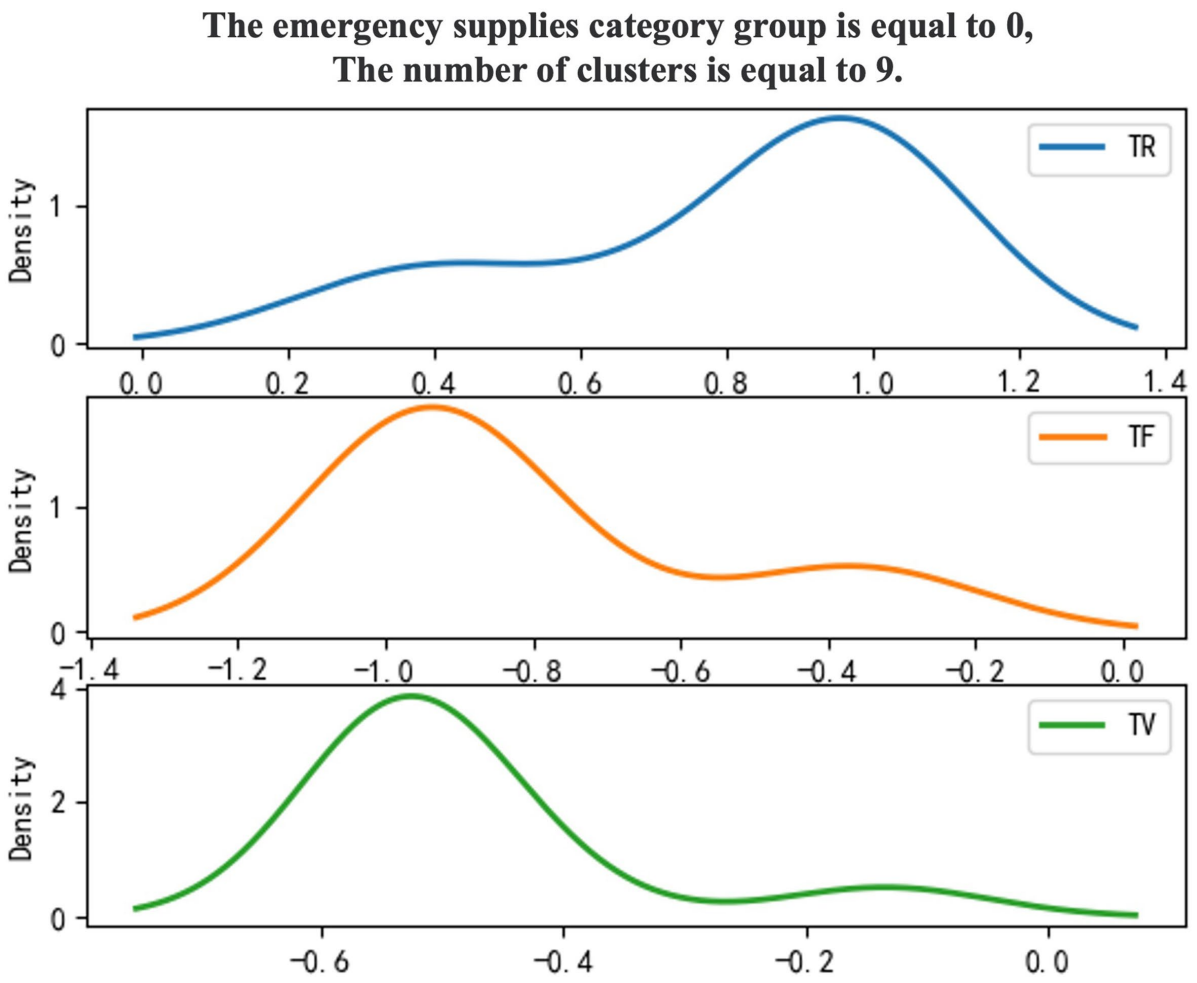
Category 0 emergency supplies.

**Figure 2:**
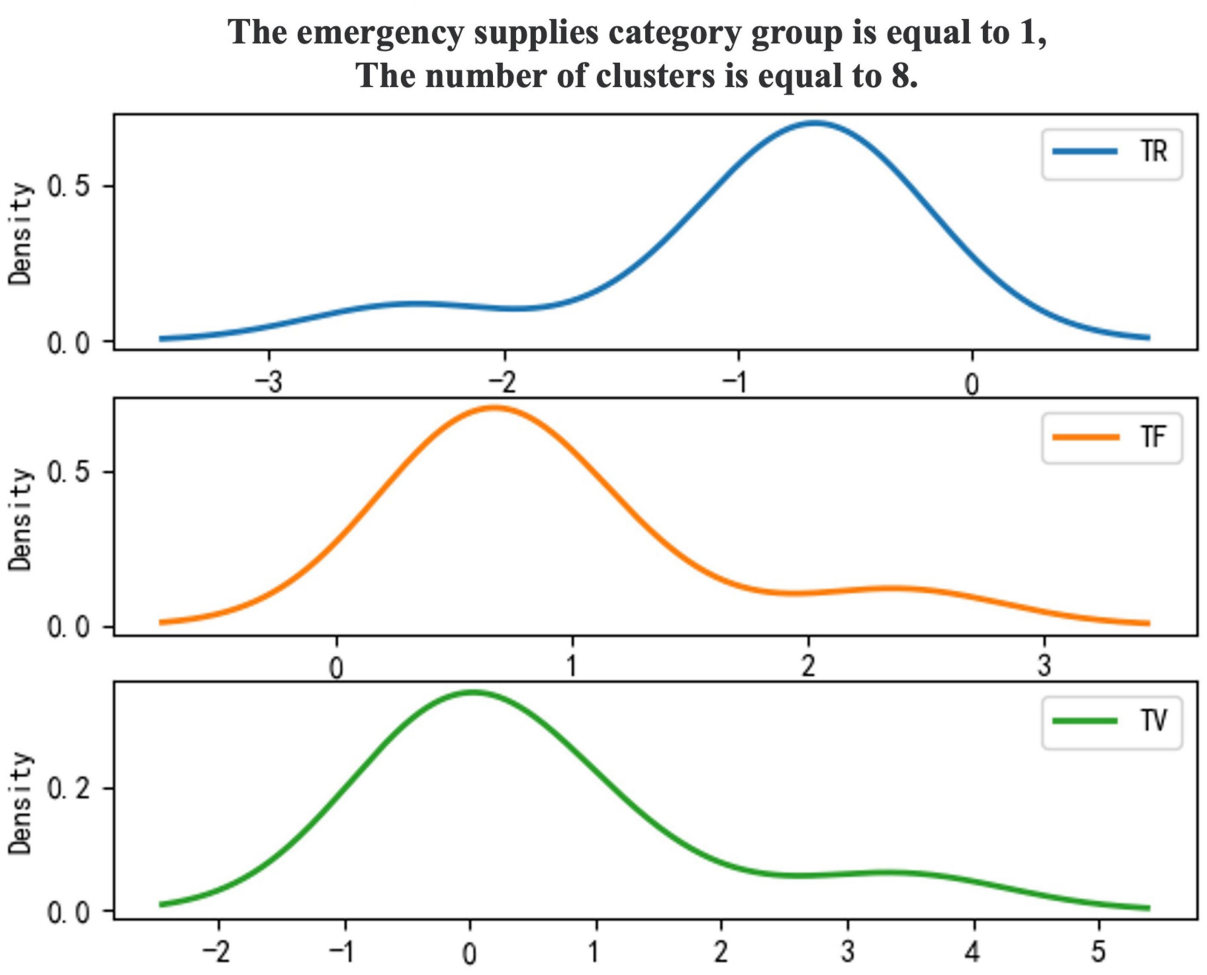
Category 1 emergency supplies.

Use 0–1 to mark emergency supplies categories, and the density diagram of the material import clustering results (Figures 1, 2).

**Figure 3:**
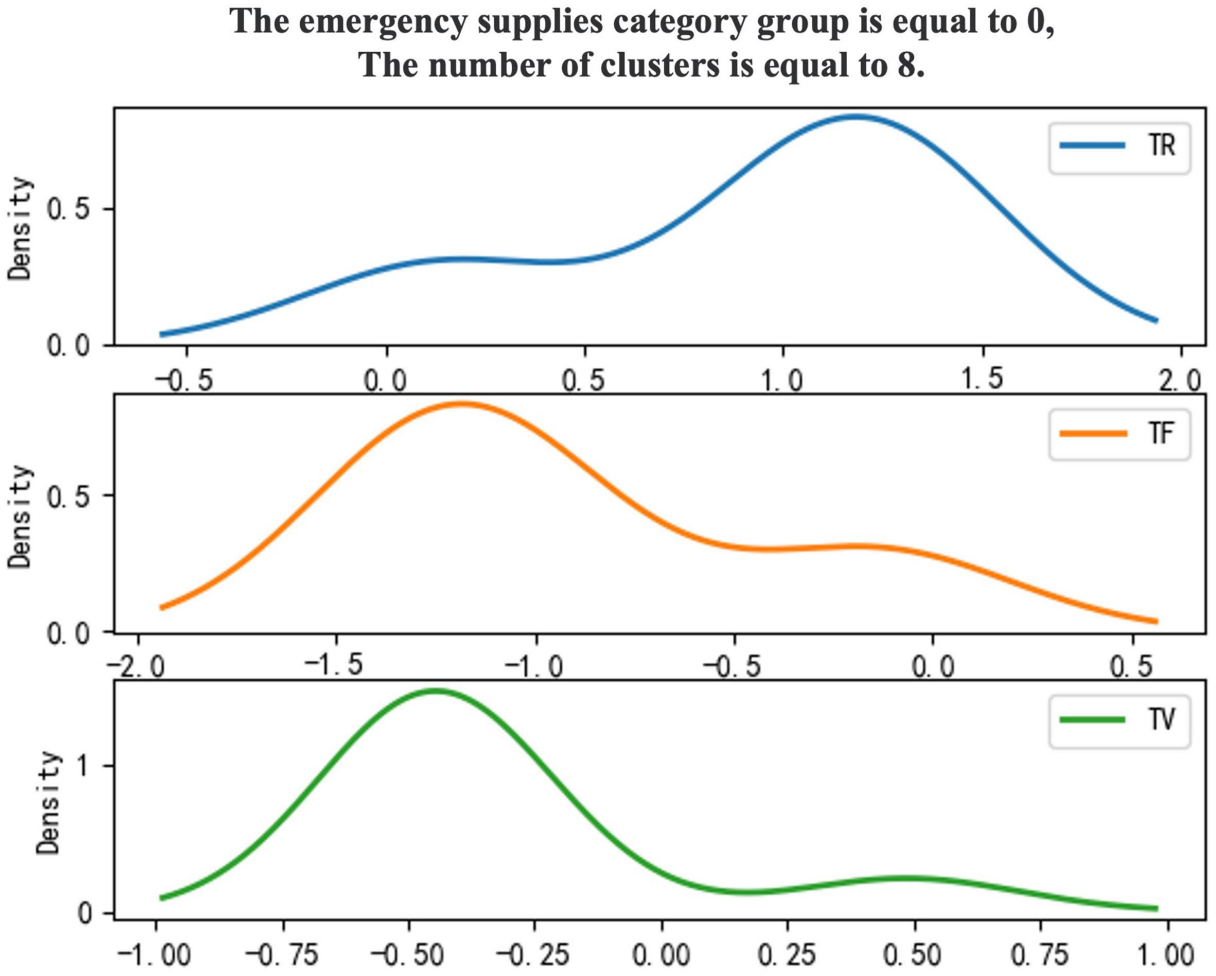
Category 0 emergency supplies.

**Figure 4:**
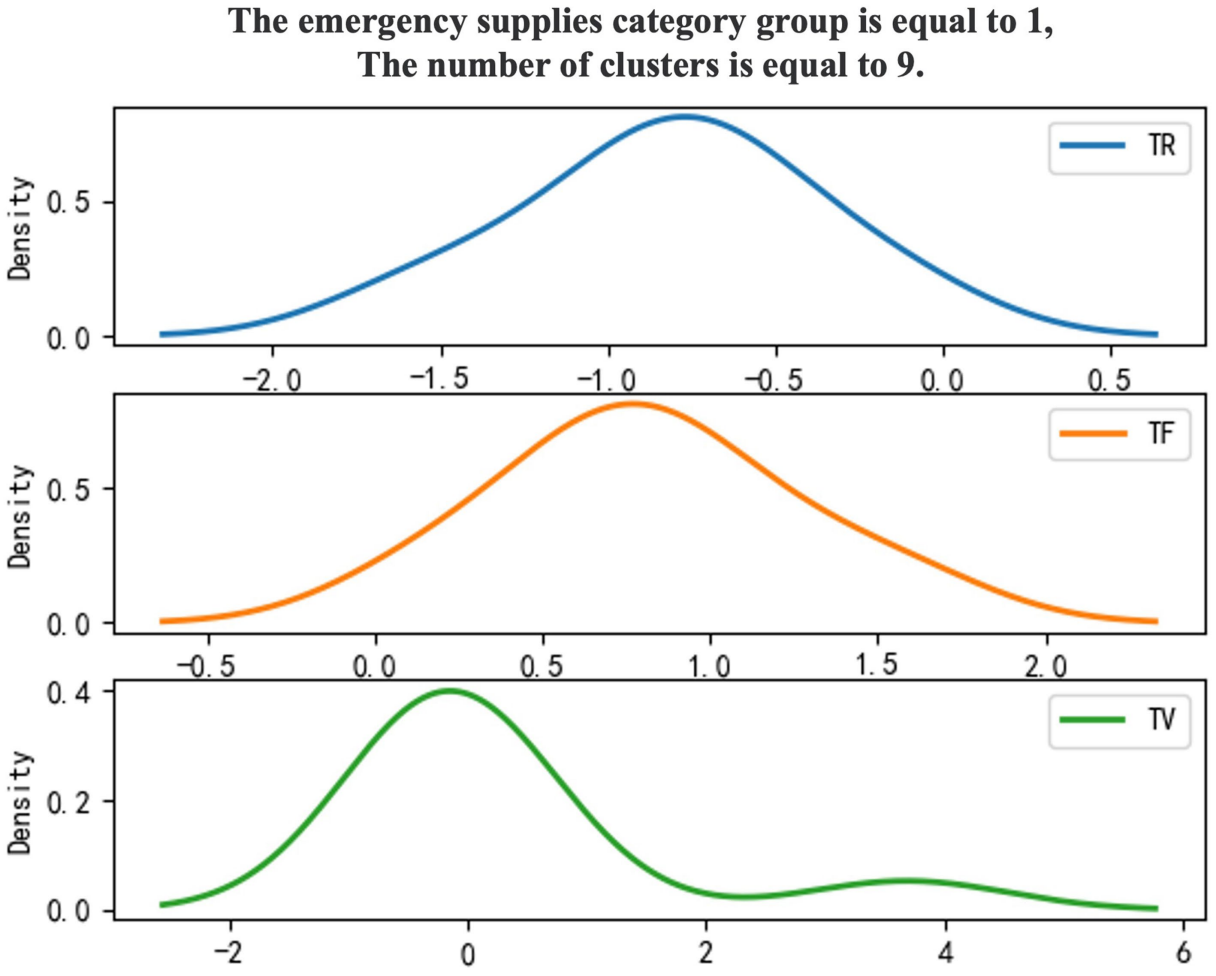
Category 1 emergency supplies.

Use 0–1 to mark emergency supplies categories, and the density diagram of the material export clustering results (Figures 3, 4).

### 4.4 Mark emergency supplies category

To enhance the clarity of the analysis regarding the categories of emergency supplies, a clustering model has been employed to categorize these supplies. The characteristic results of the TR-TF-TV values derived from the import and export statistics of emergency supplies, organized by category, are presented in Table 6.

**Table 6:** Characteristics of TR-TF-TV value statistics of emergency supplies by category.

Trade type	Tag type	TR	TF	TV
Import	0	18975.22	126.5556	6.24E+08
	1	17859.25	1254.75	9.85E+09
Export	0	18331.13	1064.875	1.79E+10
	1	14899.22	4496.778	8.23E+10

## 5 Discussion and recommendations

### 5.1 Emergency supplies clustering results

The analysis of customs clearance supervision strategies for emergency supplies is divided into two main components. The first component involves clustering emergency supplies based on three model indicators that correspond to their urgency of demand. This classification reflects varying degrees of urgency associated with different emergency supplies. The second component integrates the analysis objectives to examine the characteristics of each group of emergency supplies, assessing their importance and urgency, and subsequently ranking these groups. In this context, the mean values of the three indicators, TR, TF, and TV, as presented in Table 6, are employed to determine high and low urgency levels. Values below the average are categorized as ‘low’, while those above the average are designated as ‘high’.

As illustrated in Table 7, emergency material groups are categorized and ranked based on their comparison with the mean values of model indicators. It is determined that emergency material group 0 consists of general emergency materials, while group 1 encompasses important emergency materials. The primary criteria for classification are as follows: (1) Important emergency supplies are characterized by a high TF, indicating a significant urgency of demand. Additionally, a high TV suggests a substantial trade scale, whether through imports or exports, resulting in a pronounced impact on the safety and convenience of cross-border trade. (2) General emergency supplies are identified by a high TR, reflecting a frequent occurrence of import and export activities and constituting a considerable portion of customs clearance operations.

**Table 7:** Emergency supplies clustering results and ranking.

Trade type	TR	TF	TV	Tag type	Quantity	HS Code	Supply type	Sorting
Import	high↑	low↓	low↓	0	9	30,022,000	Ordinary	2
						39,262,011		
						40,151,200		
						63,079,010		
						84,192,000		
						84,196,011		
						84,196,019		
						90,192,010		
						90,192,020		
	low↓	high↑	high↑	1	8	30,021,500	Important	1
						38,089,400		
						38,220,090		
						40,151,900		
						62,101,030		
						63,079,000		
						90,049,090		
						90,251,990		
Export	high↑	low↓	low↓	0	8	30,022,000	Ordinary	2
						38,089,400		
						40,151,200		
						63,079,010		
						84,196,011		
						84,196,019		
						90,192,010		
						90,192,020		
	low↓	high↑	high↑	1	9	30,021,500	Important	1
						84,192,000		
						38,220,090		
						40,151,900		
						62,101,030		
						63,079,000		
						90,049,090		
						90,251,990		
						39,262,011		

Table 7 illustrates that the disinfectant classified under HS Code 84192000 serves as a general emergency material in import trade activities, while simultaneously being categorized as an important emergency material in export trade activities. Conversely, the disinfectant with HS Code 38089400 is recognized as a general emergency material in import trade activities, yet it is deemed an important emergency material in export trade activities. Furthermore, medical plastic gloves classified under HS Code 39262011 have transitioned from being categorized as ordinary emergency supplies in import trade activities to being recognized as important emergency supplies in export trade activities.

**Figure 5:**
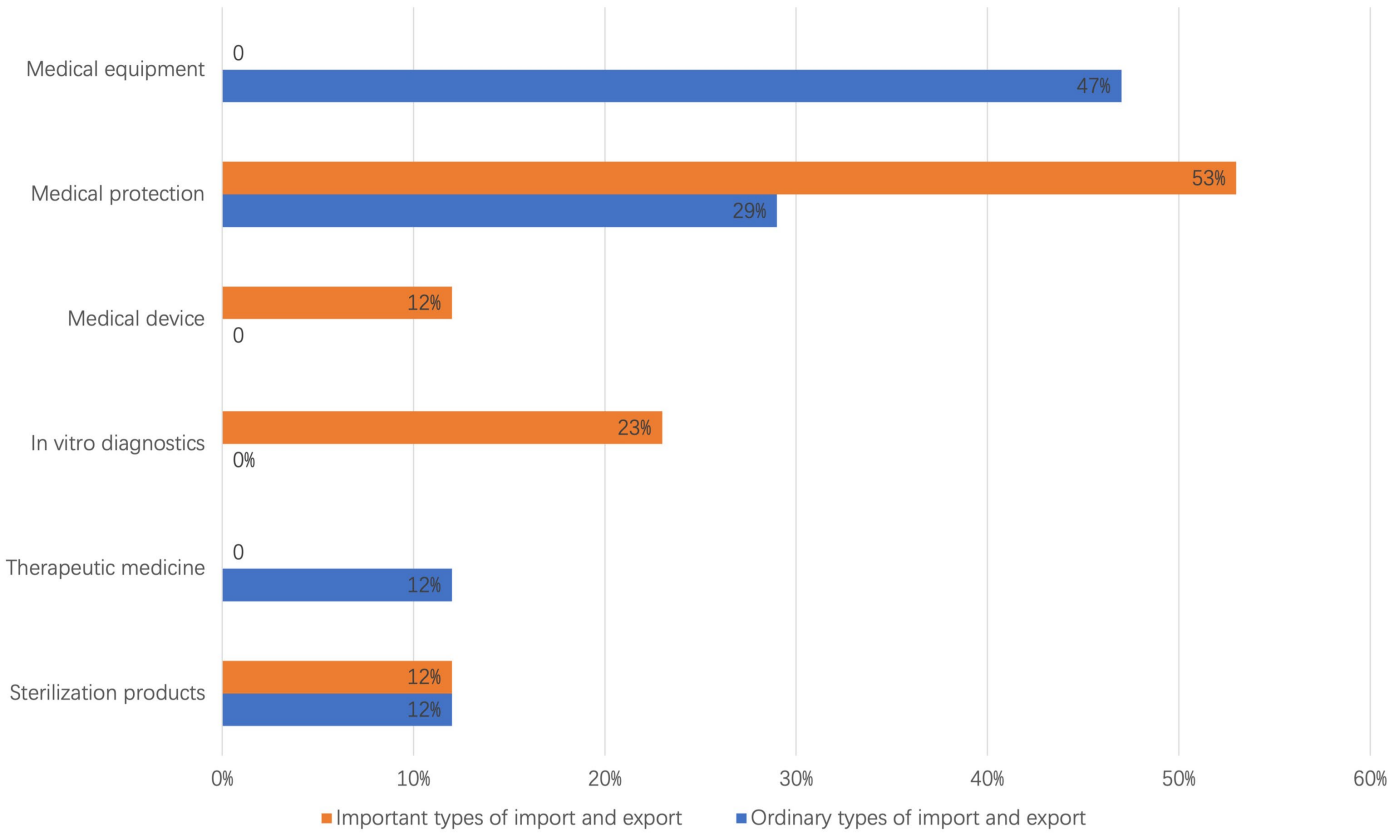
Clustering result proportion analysis.

According to the clustering results presented in Table 7, the respective proportions of six categories of emergency supplies involved in import and export trade activities, as detailed in Table 2, are illustrated in Figure 5. The categories of supplies pertinent to both the import and export of important emergency supplies and general emergency supplies include medical protection and disinfection products, which constitute two major categories. The import and export of important emergency supplies primarily encompass four categories: *in vitro* diagnostics, disinfection products, medical protection, and medical equipment. Among these, medical protection represents the highest proportion, accounting for approximately 53%. In contrast, the import and export of general emergency supplies include four major categories: therapeutic drugs, disinfection products, medical protection, and medical equipment, with medical equipment comprising the largest share at about 47%.

### 5.2 Optimization of customs clearance strategy for emergency supplies

The concept of a “gridded” unified model is essential for enhancing the customs clearance capacity for emergency supplies, serving as a vital mechanism for safeguarding public safety and national security. It is imperative to establish a “big security” perspective that systematically examines the global public security landscape and the associated risk challenges. A comprehensive understanding of one’s own prevention and control characteristics, as well as risk response strategies, is crucial for effectively managing these risks. The existing emergency management framework within the customs system must develop standards and clear directives from a macro perspective. This requires a systematic approach akin to a “national game of chess” or even a “global game of chess” to formulate emergency management indicators across the customs system, encompassing areas such as health, food, commerce, and knowledge. A holistic strategy for security prevention and control should be devised, addressing various domains including property rights, smuggling, and anti-terrorism. From a micro perspective, refined management practices should be implemented, treating customs units at all levels as distinct “grid” areas. This involves establishing and enhancing the port and territorial safety supervision chain, conducting comprehensive emergency risk inspections, standardizing labeled indicators, and promoting multifunctional integration. Additionally, a standardized emergency command system should be established to manage emergencies within these “grids” as effectively as possible. Through these measures, the transformation from “departmental security” to “overall security,” and from “self-security” to “mutual security” has been achieved, significantly enhancing the customs’ capacity to respond to various risks.

The regulatory guidelines for “grade classification” currently exhibit a lack of relevant provisions regarding “zoning and grading” or “grading and classification” in the context of emergency operations. It is essential to develop work guidelines that are reflective of “practical operation,” tailored to local practices, which will refine the management requirements across various levels of prevention and control. For example, within the framework of “two centers, three systems” established by China Customs, enhance management requirements by drawing insights from local practices. This approach promotes the integration of sectors and fosters both vertical and horizontal coordination. Additionally, it is crucial to strengthen the decision-making system for customs clearance management concerning emergency materials related to public health crises, thereby improving the rapid and effective emergency response mechanism. Furthermore, it is important to ensure a seamless connection between the General Administration and the directly affiliated customs during emergency responses. For example, the development of customs clearance support capabilities for emergency supplies should be prioritized as a critical objective of customs during the ‘14th Five-Year Plan’ period. It is essential to establish a comprehensive prevention and control command system alongside a joint prevention and control mechanism that emphasizes unified command, coordination, and interlinkage for responsive and efficient operations. Enhancing collaboration with local departments is crucial; thus, work guidelines should be formulated to reflect a ‘level and classification’ approach. Furthermore, it is important to standardize emergency response procedures and measures applicable to various public emergencies. A supervisory system that aligns with emergency management should be implemented, incorporating classified supervision based on the type, transportation method, and purpose of emergency supplies. To enhance clarity and safety in emergency management, it is essential to prominently label the outer packaging of products with the phrase “Emergency Use Only.” Additionally, implementing QR codes on general trade goods can aid in differentiating these items. Establishing safety signs and traceability mechanisms is crucial. It is also important to clarify the priority of application in instances where regulatory systems may overlap. The national integration of customs clearance for emergency supplies, along with a new “graded and classified” supervision model, designates the consignee as both the declaration and responsible subject. Building upon existing trade methods, a distinct supervision model for “emergency supplies” is proposed, which adheres to the principle of minimum necessity. This model aims to streamline declaration elements and ensure that declaration items are scientifically justified. Furthermore, the classification of commodities and product catalogs should be standardized and aligned with the national emergency support material classification.

The customs clearance of “grid classification” materials is based on the “above graded classification” supervision and the “grid management” model. This approach explores a dynamic supervision model for the customs clearance of emergency materials and aims to establish standardized management indicators throughout the entire supply chain, encompassing declaration, customs clearance, reserves, updates, allocation, and distribution. For instance, in the case of emergency materials with well-defined flows, such as donations and government purchases provided or reissued by relevant departments, unnecessary administrative licenses and approvals will be eliminated. Additionally, the primary responsibilities of recipients and local governments will be reinforced to target import and export trade activities based on the results of material clustering. For general emergency supplies, such as medical equipment and therapeutic drugs, which constitute a significant portion of the trade, it is essential to develop standardized measures. These include the creation of a material database, the construction of a material risk screening model, and the dynamic adjustment of customs clearance random inspection ratios based on the risk screening outcomes of the model. These measures aim to ensure the security of trade in emergency supplies while facilitating customs clearance. In addition to maintaining the established customs clearance supervision measures, it is essential to recognize the significant proportion of critical emergency supplies for medical protection and *in vitro* diagnostics within import and export trade activities. Therefore, we should develop specialized declaration channels for customs enterprises, reduce the frequency of random customs inspections, and ensure timely compliance with personalized customs clearance safety and facilitation measures, such as comprehensive regional coverage, to adequately meet the demand for these emergency supplies. Furthermore, provided that overseas procurement certificates and quality commitments are submitted, we should employ the ‘multi-inspection-in-one’ approach to enhance follow-up supervision based on the specific intended use of the materials. This includes considerations such as the enterprise’s own use of prevention and control materials, self-payment, and limited usage. For materials imported under general trade, the procedures will be streamlined and expedited. In conjunction with a stringent crackdown on smuggling, infringement, and other illicit activities, it is imperative to prohibit the importation of foreign waste to prevent unscrupulous individuals from exploiting regulatory loopholes for personal gain. We must enhance our awareness and capability to support the overarching goals of national diplomacy and foreign trade. While implementing essential port controls, it is crucial to adjust the level of oversight in a timely manner. A dynamic supervision mechanism should be established to respond to fluctuations in the supply and demand for emergency supplies. This mechanism should leverage technical trade measures to facilitate the expansion of exports, address domestic overproduction, and prevent substandard materials from entering the international market, thereby safeguarding our country’s political, economic, and diplomatic interests.

### 5.3 Conclusion

This article examines the typical characteristics of public health emergencies, including their complexity and cross-border nature, and systematically investigates methods to ensure the safety and efficiency of customs clearance for emergency supplies. It primarily involves the collection and organization of import and export trade data across six major categories of representative emergency supplies, Taking China Customs as a research case. By integrating K-means clustering techniques, the study develops an enhanced RFM cluster analysis model, designated as TR-TF-TV, and subsequently proposes customs supervision strategies for emergency supplies. These strategies encompass a clustering analysis of trade data to facilitate safe and efficient customs clearance, the maintenance of integrity and stability within the emergency supplies supply chain, the enhancement of the national emergency management system, and the improvement of response capabilities to public health emergencies. This analysis of trade data related to import and export emergency supplies, grounded in the improved RFM clustering model, represents an exploratory effort in original model design. Acknowledging the inherent limitations in the model’s index design and data sample selection, we aim to refine these aspects in future research, with the goal of enhancing and validating the model to further optimize related countermeasures.

## Data Availability

The original contributions presented in the study are included in the article/Supplementary material, further inquiries can be directed to the corresponding author.
